# How many mosquito nets are needed to achieve universal coverage? Recommendations for the quantification and allocation of long-lasting insecticidal nets for mass campaigns

**DOI:** 10.1186/1475-2875-9-330

**Published:** 2010-11-18

**Authors:** Albert Kilian, Marc Boulay, Hannah Koenker, Matthew Lynch

**Affiliations:** 1 Malaria Consortium, Development House, 56-64 Leonard Street, London EC2A 4LT, UK; 2 Department of Health, Behavior & Society, Johns Hopkins Bloomberg School of Public Health, Baltimore, USA; 3 Center for Communication Programs, Johns Hopkins Bloomberg School of Public Health, Baltimore, USA

## Abstract

**Background:**

Long-lasting insecticidal nets are an effective tool for malaria prevention, and "universal coverage" with such nets is increasingly the goal of national malaria control programmes. However, national level campaigns in several countries have run out of nets in the course of distribution, indicating a problem in the method used to estimate the quantity needed.

**Presentation of hypothesis:**

A major reason for the shortfall in estimation is the mismatch between the quantification factor used to plan procurement and the allocation algorithm used at community level, in particular the effect of needing to add an additional net to households with an odd number of inhabitants. To solve this problem a revised quantification factor is suggested.

**Testing hypothesis:**

Based on data from a broad range of household surveys across Africa, the effect of odd-numbered households on numbers of nets distributed is estimated via two frequently used allocation methods. The impact of these algorithms on the proportion of households reaching a person to net ratio of 2:1, a frequently used marker of universal coverage is then calculated.

**Implications:**

In order to avoid stock-outs of nets during national coverage campaigns, it is recommended to use a quantification factor of 1.78 people per net, with an additional allocation factor suggested to account for other common problems at the community level resulting in a final recommended ratio of 1.60 people per net. It is also recommend that community level allocation procedures be aligned with procurement estimates to reduce shortages of nets during campaign distributions. These analyses should enable programme managers to make evidence-based decisions and support a more efficient and effective use of LLIN distribution campaign resources.

## Background

Insecticide treated nets (ITN) are an effective tool for preventing the transmission of malaria [1]. This is particularly the case since regular re-treatment of nets with insecticide has become unnecessary with the introduction of long-lasting insecticidal nets (LLIN) [2]. Recent efforts promoting the use of LLIN have shifted their emphasis from a focus on vulnerable populations to a broader objective of universal coverage, defined at the household level as the use of insecticide-treated nets by all household members regardless of age or gender [3]. There is an emerging consensus that a ratio of at least one LLIN for every two household members is typically sufficient to achieve universal coverage in a population [4].

Centralized mass distribution campaigns have served as the cornerstone of efforts to achieve universal coverage [5]. Recent evaluations of these campaigns support their effectiveness at broadening household ownership of LLIN, i.e. households with at least one LLIN increase substantially [6]. However, these campaigns at times fail to provide households with sufficient quantities of nets to reach the desired ratio of one LLIN per two household members [7,8]. As a consequence, the effect of these campaigns on the proportion of all people sleeping under a LLIN often falls short of target levels.

Inadequate procurements may be one reason that these campaigns fail to provide households with a sufficient number of nets. The World Health Organization/Global Malaria Programme (WHO/GMP) recommends dividing the estimated total population by a factor of two when calculating the total number of nets needed to achieve the desired ratio of 1 net per every two people [4]. In practice, this approach tends to underestimate the overall need for nets. In 2009, a pilot distribution campaign in Diébougou district, Burkina Faso estimated the total number of nets required for the campaign to be 56,191, based on the WHO approach plus a 10% margin of error. However, a subsequent registration of all sleeping places identified a total need of 67,404 nets, a 32% increase from the initial estimate [9]. A distribution campaign by the Peace Corps in Velingara District, Senegal used the WHO approach, minus the nets distributed the prior year to children under five years of age, to estimate that 70,486 LLIN would be sufficient to achieve universal coverage in the district. During the campaign, when nets were allocated to households based on the number of sleeping places, implementers learned that they needed a total of 115,619 LLIN, a 64% increase from the initial estimate (personal communication: Debbie Gueye, United States Agency for International Development (USAID) Senegal*)*. A similar campaign in Uganda noted that the actual number of nets required for universal coverage was 30% greater than the estimate obtained using the WHO calculation (personal communication: Kojo Lokko, AFFORD Project).

Inefficient household allocation strategies may be a second factor limiting the ability of these campaigns to achieve universal coverage. Countries use a variety of approaches for allocating nets to households during their distribution campaigns. In some countries, campaigns distribute a fixed number of nets, typically two or three nets, to each household. In other countries, the number of nets allocated to a household varies by the size of the household or by the number of sleeping places in the household. This variation in approaches illustrates the lack of clarity in the current understanding regarding how best to allocate nets in support of universal coverage objectives.

Drawing on existing household survey data, this paper provides empirical support for an improved algorithm for estimating the number of LLIN needed to achieve universal coverage within a given population and evaluates the various approaches for allocating LLIN to specific households. These analyses should provide guidance to programme managers to make evidence-based decisions and support a more efficient and effective use of LLIN distribution campaign resources.

### Presentation of the hypotheses

#### Quantifying the procurement of LLIN

The current algorithm for calculating the number of LLIN necessary to provide one net for every two people in a population simply divides the total population size by a factor of two [4]. However, since an LLIN is indivisible and incapable of being shared between two different households, this approach systematically underestimates the demand in households with an uneven number of household members. For example, two three-person households would have a total population of six. Based on the current approach of dividing this population by two, managers would procure three LLIN to achieve universal coverage in these two households. In practice, however, one household would receive two nets with only one net remaining for the second household and the campaign would only achieve universal coverage in one of the two households.

A more accurate algorithm would use a population-level divisor that accounts for the one additional LLIN required by households with an uneven number of members. To identify this divisor and assess the additional coverage that would result from its use, two scenarios were used to simulate the allocation of LLIN to households sampled in 12 Demographic and Health Surveys (DHS) [10] and six additional sub-national household-based surveys [[7,8,11], unpublished data, Malaria Consortium). In both scenarios, households with an even number of *de jure *members (i.e. all those who usually live there but excluding temporary visitors) received a quantity of nets equal to half of the household size. The number of nets allocated to households with an uneven number of *de jure *members differed between the two scenarios. In Scenario A, households received a quantity of nets equal to half of the household size minus 1; while in Scenario B, households received a quantity of nets equal to half of the household size plus 1. Table 1 provides the exact calculations used as allocation rules for the two scenarios.

**Table 1:** Simulated allocation rules for Scenario A and Scenario B

	Number of LLINs allocated to a household	
	**Even number of *de jure *household members**	**Uneven number of *de jure *household members**

Scenario A	$\frac{Number\;of\;HH\;members}{2}$	$\frac{\left(Number\;of\;HH\;members-1\right)}{2}$

Scenario B	$\frac{Number\;of\;HH\;members}{2}$	$\frac{\left(Number\;of\;HH\;members+1\right)}{2}$

Once households are allocated a quantity of nets according to the rules described in Table 1, the ratio of household members to allocated nets can be calculated for each household and averaged across the entire sample. This average, labelled as the mean number of household members per allocated net, is equivalent to the population-level divisor used to estimate the total number of nets required for a campaign. This divisor calculated under Scenario A could be used to calculate the total number of nets needed for a campaign that did not plan to provide an additional net to households with an uneven number of members. Under Scenario B, this divisor could be used to calculate the number of nets needed for a campaign that did plan to provide an additional net to households with an uneven number of members.

#### Evaluating net allocation strategies

Strategies to allocate nets to households are guided by two primary questions. First, should the campaign allocate a fixed or varying number of nets to each household? And, if households should receive a varying number of nets, should the number of nets allocated to the household be based on the number of people or the number of sleeping places within the household? At present, there is little empirical data upon which program managers can base their decisions to these questions.

The decision to allocate a fixed number of nets to each household seems based on the assumption that a single quantity can be identified that provides a substantial number of households with the correct number of nets, while minimizing the proportion of households that receive too many nets or too few nets. To test this assumption, the results of two fixed allocation campaigns, one providing two nets to each household and the other providing three nets, were assessed using the distribution of household sizes in the 18 datasets. Households were classified as receiving the correct number of nets if they received one net for every two people in the household (3-4 household members for the two-net campaign; 5-6 members for the three-net campaign). They were classified as receiving too many nets if they had fewer household members and were classified as receiving too few nets if they had more household members.

When the decision is made to vary the number of nets allocated to a household, the number of sleeping places within the household appears to be the logical metric to use to ensure that a mosquito net covers every person sleeping in the household. In practice, this approach presents some concerns. First, where individuals sleep on a variety of surfaces, the definition of a sleeping place may include areas in which several people sleep and that may be too large to be enclosed by a single net. Second, households from lower wealth quintiles may be more likely to have more individuals sharing fewer sleeping places. Use of sleeping places to allocate nets then may result in an inequitable distribution of nets favouring households from higher wealth quintiles. To investigate these concerns, data from five surveys that measured both the number of household members and the number of sleeping places were analysed.

### Testing the hypothesis

#### What divisor will provide the correct number of LLIN to achieve universal coverage?

Recent experience suggests that dividing the total population by two underestimates the total number of LLIN required to achieve universal coverage, defined as at least one net for every two people in a household. To test the hypothesis that this underestimate is attributed to the additional net required by households with an uneven number of members, simulated allocations of LLIN to households either ignored this additional need (Scenario A) or met this additional need (Scenario B). It was expected that Scenario B would be more likely than Scenario A to reach the household threshold of universal coverage and that the mean number of persons per net in Scenario B would serve as a more effective population divisor for calculating the total number of nets needed to reach universal coverage.

Table 2 presents the results from the simulated allocation of nets under Scenario A, in which households with an uneven number of *de jure *members received a quantity of nets equal to half of the household size minus 1. Following this allocation rule, only 50 to 60% of households would receive at least one net for every two people and meet the threshold required for universal coverage. The mean number of persons per net ranged from 2.02 to 2.27, with a median score of 2.19. This "LLIN allocation factor" is slightly higher than the number (2.0) currently recommended by WHO, suggesting that the current approach should result in approximately 60-70% of households reaching the threshold of one net for every two people that is required for universal coverage.

**Table 2:** Results from simulated allocation of LLINs using Scenario A allocation rule

Country & year	% urban	Mean HH size	Nets per HH		Number of persons per net		% HH with net for two people*
				
			Mean	95% CI	Mean	95% CI	
**DHS surveys**							

Benin 06	40.4	5.04	2.36	2.32, 2.40	2.15	2.14, 2.16	56.7

Ethiopia 05	14.4	5.03	2.31	2.28, 2.34	2.22	2.21, 2.23	53.5

Ghana 08	47.8	3.74	1.81	1.78, 1.84	2.02	2.01, 2.03	64.3

Mali 06	30.5	5.68	2.64	2.59, 2.68	2.20	1.19, 2.22	53.3

Malawi 04	16.6	4.38	2.02	1.99, 2.04	2.20	2.19, 2.21	56.5

Nigeria 08	35.5	4.42	2.11	2.09, 2.14	2.05	2.04, 2.07	61.5

Niger 06	17.0	6.08	2.82	2.77, 2.88	2.21	2.20, 2.22	52.0

Rwanda 05	15.0	4.57	2.10	2.07, 2.12	2.22	2.21, 2.23	55.6

Senegal 06	46.5	9.38	4.49	4.29, 4.70	2.10	2.08, 2.12	54.6

Tanzania 07/08	24.8	4.99	2.32	2.25, 2.38	2.18	2.16, 2.19	56.2

Uganda 06	15.7	4.96	2.33	2.29, 2.37	2.13	2.12, 2.14	58.2

Guinea 05	28.5	6.09	2.85	2.78, 2.91	2.18	2.17, 2.19	54.8

**Other surveys**							

Mozambique 07 (MIS)	24.1	4.85	2.22	2.17, 2.28	2.23	2.21, 2.24	54.8

Nigeria Kano 09	24.0	4.59	2.13	2.00, 2.25	2.18	2.12, 2.25	59.0

Nigeria Anambra 09	49.3	4.41	2.04	1.97, 2.11	2.18	2.14, 2.23	59.6

Sudan NBeG 09	0	5.76	2.65	2.56, 2.74	2.20	2.17, 2.23	52.3

Uganda Adjumani 07	0	5.76	2.63	2.53, 2.74	2.27	2.24, 2.30	50.4

Uganda Jinja 07	0	6.54	3.03	2.92, 3.15	2.22	2.20, 2.25	52.3

**Figure 1:**
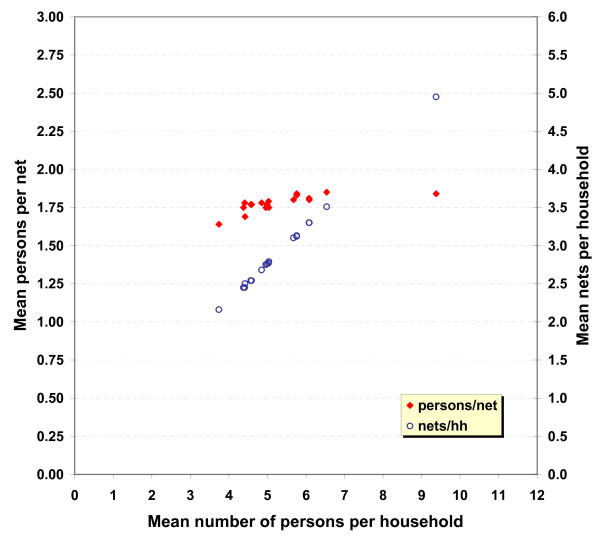
Correlation of the ratio of persons to net obtained with odd number correction and mean household size (red filled diamonds) compared to that between mean nets per household (same allocation rule) and mean household size (blue open circles)

The results from the Scenario B simulation are presented in Table 3. As expected, when households with an uneven number of *de jure *members are allocated an additional net to accommodate the extra person, all households meet the threshold of one net for every two people that is required for universal coverage. In this scenario, the mean number of persons per net ranged from 1.64 to 1.85, with a median score of 1.78. This indicates that dividing the total population by 1.78 would provide enough nets for a campaign to provide each household with at least one net for every two people in the household. The mean number of persons per net in Scenario B remains remarkably consistent across countries, regardless of the level of urbanization and the mean household size in each country (Figure 1). This suggests that a single population-derived "LLIN allocation factor" can be applied consistently across all countries.

**Table 3:** Results from simulated allocation of LLINs using Scenario B allocation rule

Country & year	% urban	Mean HH size	Nets per HH		Number of persons per net		% HH with net for two people*
				
			Mean	95% CI	Mean	95% CI	
**DHS surveys**							

Benin 06	40.4	5.04	2.79	2.75, 2.83	1.75	1.74,1.75	100

Ethiopia 05	14.4	5.03	2.77	2.74, 2.81	1.79	1.78, 1.79	100

Ghana 08	47.8	3.74	2.16	2.13, 2.20	1.64	1.64, 1.65	100

Mali 06	30.5	5.68	3.10	3.05, 3.15	1.80	1.79, 1.80	100

Malawi 04	16.6	4.38	2.45	2.42, 2.48	1.75	1.75, 1.76	100

Nigeria 08	35.5	4.42	2.50	2.47, 2.53	1.69	1.68, 1.69	100

Niger 06	17.0	6.08	3.30	3.24, 3.37	1.81	1.81, 1.82	100

Rwanda 05	15.0	4.57	2.54	2.52, 2.56	1.77	1.77, 1.78	100

Senegal 06	46.5	9.38	4.95	4.74, 5.16	1.84	1.83, 1.86	100

Tanzania 07/08	24.8	4.99	2.76	2.69, 2.82	1.77	1.76, 1.78	100

Uganda 06	15.7	4.96	2.75	2.70, 2.79	1.75	1.74, 1.76	100

Guinea 05	28.5	6.09	3.30	3.23, 3.36	1.80	1.80, 1.81	100

**Other surveys**							

Mozambique 07 (MIS)	24.1	4.85	2.68	2.62, 2.74	1.78	1.77, 1.79	100

Nigeria Kano 09	24.0	4.59	2.54	2.44, 2.63	1.77	1.74, 1.80	100

Nigeria Anambra 09	49.3	4.41	2.45	2.37, 2.52	1.78	1.75, 1.80	100

Sudan NBeG 09	0	5.76	3.12	3.03, 3.22	1.83	1.81, 1.85	100

Uganda Adjumani 07	0	5.76	3.13	3.02, 3.23	1.84	1.82, 1.85	100

Uganda Jinja 07	0	6.54	3.51	3.40, 3.63	1.85	1.84, 1.87	100

These results are nearly identical to those obtained from intensive micro-census investigations in Mozambique and Cambodia. In Mozambique, information on the number, gender, and age of household members was used to determine the likely sleeping arrangements of the household members and identified that 1.75 persons per net was necessary to achieve universal coverage [12]. A similar figure of 1.79 was found in a micro-planning exercise in Cambodia, where a census was done in approximately 1,000 households and nets allocated individually with odd numbered households rounded up (personal communication, Steve Mallor, Malaria Consortium Cambodia).

#### What is the most effective way to allocate nets to households to achieve universal coverage?

The second hypothesis examined the relative effectiveness of using a fixed allocation of nets to achieve universal coverage. It was expected that the variation in household sizes would limit the effectiveness or efficiency of this approach, suggesting the use of tailored allocations as the recommended approach for determining the number of nets to be given to households.

As expected, allocating a fixed number of LLIN to each household does not appear to be an effective approach for achieving universal net coverage or an efficient way to allocate nets to households (Table 4). When two nets are allocated to households, the percent of households receiving one net for every two household members ranges from a low of 11.3% to a high of 35.0%. When three nets are allocated, the percent of households receiving one net for every two household members ranges from 15.7% to 43.3%. In nearly all countries, an allocation of two nets per household provides an insufficient number of nets to achieve universal coverage, while an allocation of three nets provides households with too many nets and is an inefficient use of resources.

**Table 4:** Effects of a fixed household allocation of two or three nets with respect to the universal coverage criteria "1 net for 2 people"

Country	Two nets provided per HH			Three nets provided per HH		
	
	Too many nets	Just right	Too few nets	Too many nets	Just right	Too few nets
**DHS**						

Benin 06	20.1	29.2	50.7	49.3	25.8	24.9

Ethiopia 05	14.1	29.5	56.4	43.6	30.8	25.6

Ghana 08	37.0	29.4	33.5	66.5	20.8	12.7

Mali 06	14.0	27.3	58.7	41.3	25.6	33.1

Malawi 04	21.0	26.1	42.9	57.1	26.2	16.7

Nigeria 08	30.7	26.8	42.6	57.4	21.8	20.8

Niger 06	11.2	25.6	63.2	36.8	26.7	36.4

Rwanda 05	17.7	35.0	47.3	52.7	28.8	18.5

Senegal 06	8.8	11.3	79.9	20.1	15.7	64.2

Tanzania 07/08	19.3	29.1	51.6	48.4	26.5	25.1

Uganda 06	20.9	26.0	53.0	47.0	25.9	27.1

Guinea 05	12.5	24.3	63.2	36.8	25.9	37.3

**Other surveys**						

Mozambique 07 (MIS)	16.0	33.8	50.2	49.8	28.6	21.6

Nigeria Kano 09	19.6	33.1	47.3	52.7	30.3	17.0

Nigeria Anambra 09	23.1	31.5	45.4	54.6	28.3	17.1

Sudan NBeG 09	5.2	17.9	76.9	23.1	43.3	33.6

Uganda Adjumani 07	1.6	29.7	68.7	31.3	36.8	31.9

Uganda Jinja 07	1.7	23.2	75.1	24.9	27.8	47.3

Key:

2 nets to each HH: Too many (1-2 de jure household members); Just right (3-4 members); Too few (5+ members)

3 nets to each HH: Too many (1-4 de jure household members); Just right (5-6 members); Too few (7+ members)

Since this suggests allocating a varied, rather than fixed, number of nets to households, the remaining question concerns the use of household members or sleeping places to determine the amount of nets required by each household. It was hypothesized that the subjective definition of a "sleeping place" may result in an identification of spaces in which multiple individuals sleep and which may be too large to be covered by a single LLIN. It was further hypothesized that the number of sleeping places in a household would be positively related to household wealth and that allocating LLIN by sleeping places would disproportionately favour the distribution of nets to wealthier individuals.

**Figure 2:**
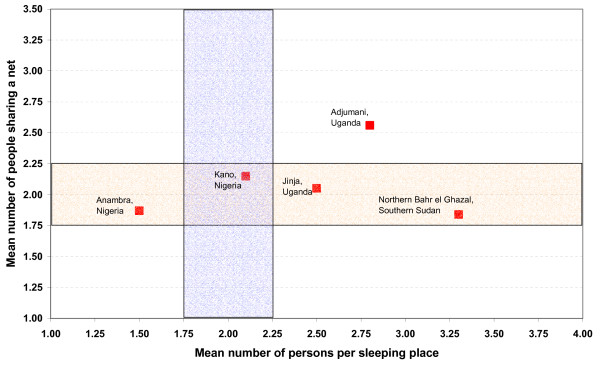
**Correlation between mean number of net users (if net used) and mean number of people per sleeping place**. Ideally all values should fall into the intersection of the two boxes.

Figure 2 presents data from five surveys that included a measurement of sleeping places in the household. As this figure illustrates, the variation of people per sleeping place was much wider than the variation of people per net. The ratio of people per sleeping place across these five surveys ranged from 1.5 to 3.3, while the mean number of people sharing a net remained between 1.75 and 2.25 in four of the five surveys. The most likely reason for the increased variation in people per sleeping place is the definition of sleeping places that, in some cases, are not equivalent to a space in which people could share a net. Distribution campaigns that allocate nets to households based on their sleeping places may undersupply households in which the number of people per sleeping place is lower than 2.0 and may oversupply households in which the number of people per sleeping place is higher than 2.0.

**Figure 3:**
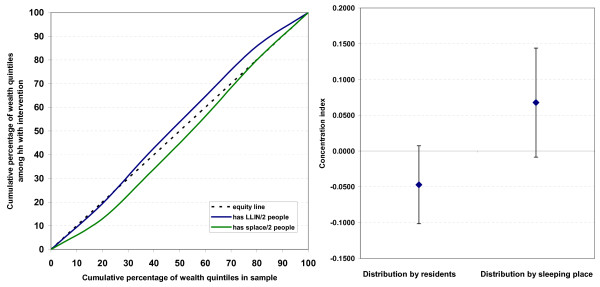
**Concentration curve and index comparing the equity of universal coverage in Northern Bahr el Ghazal, Southern Sudan, between the actual distribution by residents and the distribution had one net been given per sleeping place (N = 502)**.

Data from Northern Bahr el Ghazal in Southern Sudan (unpublished data: Malaria Consortium, see annex for details on survey and methodology [Additional file 1]) suggest that the use of sleeping places to allocate LLIN may result in an unequal distribution of nets favouring wealthier households. A post-campaign survey showed that the mean number of sleeping places increased with wealth quintile while the mean number of *de-jure *residents did not, resulting in a decreasing mean number of persons per sleeping place with increasing wealth (Table 5). Using the concentration curve and index for "universal coverage" to assess equity of LLIN distribution showed that the actual distribution was equitable and even somewhat pro-poor with a concentration index of -0.047 (95% CI -0.101, 0.008) (Figure 3). In contrast, the concentration curve for sleeping places, i.e had one net be given for each sleeping place, showed a slightly pro-rich effect on universal coverage with an index of 0.068 (95%CI -0.008, 0.144). In this case the difference did not quite reach the p < 0.05 statistical significance level but this is merely a question of sample size. The example demonstrates that the use of sleeping places for net allocation can indeed have some undesirable equity effects.

**Table 5:** Distribution of residents and sleeping places by wealth quintile in Northern Bahr el Ghazal, Southern Sudan

Wealth quintile	Mean Persons per household	Mean sleeping places per household	Mean Persons per sleeping place
Lowest	5.56	1.40	4.47

Second	5.19	1.73	3.43

Third	5.73	2.10	3.04

Fourth	5.76	2.42	2.62

Highest	6.43	2.42	2.97

**Total**	**5.75**	**2.03**	**3.29**

### Implications of the hypothesis

These results recommend revising the current approach for calculating the number of LLIN necessary for achieving universal coverage through a centralized mass distribution campaign. Rather than dividing the total population by a factor of 2, a new divisor of 1.78 is necessary to account for the additional LLIN needed in households with an uneven number of members. This revised divisor is very similar to that suggested by "bottom up" approaches of micro-planning at the household level.

It should be noted, however, that these simulations are unable to account for some additional logistical hurdles experienced by distribution campaigns. First, campaigns often need to pre-position stocks of LLIN to adjust for inconsistencies between the census estimates used for planning and the actual numbers obtained during registration exercises. Second, since LLIN are packaged in bales of 50 or 100 and are difficult to transport once opened, campaigns typically round-up the number of LLIN sent to the distribution points to the nearest bale. Both practices result in a certain number of LLIN being "stuck" in the distribution chain, resulting in an additional demand of 5% to 10% of initial estimates.

The obvious solution is to include a buffer in the quantification that will compensate for these logistical "losses." While there is little empirical information regarding the extent of this margin, reasonable expectations suggest it is between 5% and 15% of the total need. Applying these rates to the recommended population divisor of 1.78 results in the corrected factors presented in Table 6.

**Table 6:** Possible further correction of the "LLIN allocation factor" to compensate for logistics (distribution chain) and variations on estimation parameters such as population growth

Correction factor	"LLIN allocation factor"
1.78 +5%	1.69

1.78 +10%	1.60

1.78 +15%	1.51

### Based on all these considerations, the authors recommend that programme managers divide their population by a factor of 1.60 to calculate the number of LLIN that they need to procure to achieve universal coverage of one net for every two people

These results also recommend allocating LLIN to households based on their household size, without an upper limit for larger households, rather than using a fixed number of nets per household. Using the number of household members, rather than sleeping places, appears more likely to provide households with a sufficient number of nets to achieve universal coverage and to result in a more equitable distribution of LLIN across all wealth quintiles. However, if sleeping places are to be used to determine the number of nets required by a household, efforts should be made to confirm that the mean number of people per sleeping place is close to 2.0 and the definition of a sleeping place needs to be clearly articulated and standardized. One possible approach would be to define sleeping places as "a place where people sleep and that can be covered by a single net."

Whether household size or sleeping places are used to allocate nets to households, a similar metric should be used to quantify the total number of nets needed for the campaign. If the method of quantification differs from that used for actual net allocation during the campaign, the allocation of nets may require a greater quantity than were estimated by the quantification method. In Kano, Nigeria, LLIN needs were quantified at two nets per household using the standard definition of "people eating from the same pot," but were allocated using the household definition of "wife with her dependents" in polygamous families. Instead of delivering two nets per household, the outcome of the post-campaign survey showed that only 1.7 nets were delivered among those who attended the distribution, with 28% of households receiving only one net [8]. In Senegal, overall need was calculated based on the ratio of one net for two people and then allocated by sleeping place during house-to-house registration. Since the average number of people per sleeping place was 1.75, a 25% deficit in nets is expected [13].

## Competing interests

The authors declare that they have no competing interests.

## Authors' contributions

AK drafted the manuscript, provided data form the sub-national studies and did part of the statistical analysis. MB provided the statistical analysis of the DHS data and contributed to the manuscript development. HK provided input on structure and campaign logistics, and ML helped draft and edit the manuscript. All authors read and approved the final manuscript.

## Authors' information

AK is director monitoring & evaluation for the Malaria Consortium; MB is Assistant Professor at the Department of Health, Behavior & Society, Johns Hopkins Bloomberg School of Public Health; HK is senior program officer at the Center for Communication Programs, Johns Hopkins Bloomberg School of Public Health; ML is Director of the Global Program on Malaria at the Center for Communication Programs, Johns Hopkins Bloomberg School of Public Health

## Supplementary Material

Additional file 1 **Net tracking survey Northern Bahr el Ghazal, Southern Sudan**. Details of the study design, methodology and analysis Click here for file
