# Supplementary material for: How many mosquito nets are needed to achieve universal coverage? Recommendations for the quantification and allocation of long-lasting insecticidal nets for mass campaigns
Source: Malar J. 2010 Nov 18;9:330. doi: 10.1186/1475-2875-9-330 (PMC2994892; doi:10.1186/1475-2875-9-330)
Supplement: Additional file 1 — Net tracking survey Northern Bahr el Ghazal, Southern Sudan. Details of the study design, methodology and analysis [file 1475-2875-9-330-S1.DOC]

**Net tracking survey Northern Bahr el Ghazal, Southern Sudan**

The survey was undertaken by Malaria Consortium in collaboration with the Ministry of Health, Government of Southern Sudan, between August 2 and September 24 2009. Study area were three counties in Northern Bahr el Ghazal State, Aweil North, Aweil West and Aweil Central where between December 2008 and April 2009 a LLIN distribution campaign was implemented. The LLIN distributions targeted all households and delivered between 1 and three LLIN depending on household size.

Survey methodology

The sampling method for the survey was community-based using the most recent data set from National Immunization Days as sampling frame. Thirty clusters (villages) were selected using probability proportional to size sampling, across the three counties with no further stratification. Then, 17 households within each cluster were selected using simple random sampling. For the second stage, a sampling frame listing all households was obtained from the village head. Then, the required number of households was randomly selected from that list. Of the 510 targeted household interviews 502 (98.4%) were completed.

Data analysis

Data were double entered in the database, using EpiData 3.1 software (The EpiData Association “Odense”, Denmark). Discrepancies were identified and corrected. The Stata statistical software (version 11, Stata Corp., USA 2009) was used for data management and analysis. Data were cleaned and checked for consistency. All analysis accounted for the survey design, adjusting for cluster sampling. Standardised sampling weights were calculated. This was done using the “svy” command family in Stata. Socio economic disparities were investigated based on asset-based wealth index obtained from Principle Component Analysis where the first component was used as the index value. Asset variables were dichotomized or scaled. The number of wives was included in the analysis as it seemed to be associated with wealth. The data set was then divided into five equal groups representing the wealth quintiles.

Concentration curves were obtained by plotting the cumulative distribution of wealth quintiles in the data set against the cumulative distribution of wealth quintiles in the sub-population of interest (here households with at least one LLIN for every two persons). Concentration indices, their standard errors and confidence intervals were calculated using the formula suggested by Kakwani et al. [1].

Reference

1. Kakwani NC, Wagstaff A, van Doorslaer E: **Socioeconomic inequalities in health: measurement, computation, and statistical inference**. *J Econometrics* 1997, **77**:87-103.
